# “I can’t read and don’t understand”: Health literacy and health messaging about folic acid for neural tube defect prevention in a migrant population on the Myanmar-Thailand border

**DOI:** 10.1371/journal.pone.0218138

**Published:** 2019-06-13

**Authors:** Mary Ellen Gilder, Pru Moo, Ahmar Hashmi, Norda Praisaengdet, Kerry Wai, Mupawjay Pimanpanarak, Verena I. Carrara, Chaisiri Angkurawaranon, Wichuda Jiraporncharoen, Rose McGready

**Affiliations:** 1 Shoklo Malaria Research Unit, Mahidol-Oxford Tropical Medicine Research Unit, Faculty of Tropical Medicine, Mahidol University, Mae Sot, Thailand; 2 Department of Family Medicine, Faculty of Medicine, Chiang Mai University, Chiang Mai, Thailand; 3 Department of Medicine, Swiss Tropical and Public Health Institute, Basel, Switzerland; 4 Centre for Tropical Medicine and Global Health, Nuffield Department of Medicine, University of Oxford, Oxford, United Kingdom; Bielefeld University, GERMANY

## Abstract

Health literacy is increasingly recognized as an important determinant of health outcomes, but definition, measurement tools, and interventions are lacking. Conceptual frameworks must include both individual and health-systems domains which, in combination, determine an individual’s health literacy. Validated tools lack applicability in marginalized populations with very low educational levels, such as migrant worker communities on the Myanmar-Thailand border. We undertake a comprehensive health literacy assessment following a case study of a recent public health campaign promoting preconceptual folic acid uptake in this community. A mixed-methods design utilized quantitative analysis of the prevalence and predictors of low Health literacy, and focus group discussions to gather qualitative data from women about proposed and actual posters used in the campaign. Health literacy was measured with a locally developed tool that has been used in surveys of the population since 1995. Health literacy was low, with 194/525 (37.0%) of tested women demonstrating adequate health literacy, despite 63.1% (331/525) self-reporting being literate. Only one third of women had completed 4th grade or above and reported grade level attained in school was more predictive of health literacy than self-reported literacy. Focus group discussions revealed that low literacy, preconceived associations, and traditional health beliefs (individual domain) interacted with complex images, subtle concepts, and taboo images on posters (health-systems domain) to cause widespread misunderstandings of the visuals used in the campaign. The final poster still required explanation for clarity. Low health literacy is prevalent among pregnant women from this migrant community and barriers to communication are significant and complex. Public health posters need piloting prior to implementation as unanticipated misperceptions are common and difficult to overcome. Verbal communication remains a key method of messaging with individuals of low health literacy and educational system strengthening and audiovisual messaging are critical for improvement of health outcomes.

## Introduction

Low health literacy (HL) is associated with adverse health outcomes across many health domains and contexts [1]. However, there is no consensus about its definition, optimal tools of measurement, or interventions [2–7]. In 1998, the World Health Organization (WHO) defined HL as “the cognitive and social skills which determine the motivation and ability of individuals to gain access to, understand and use information in ways which promote and maintain good health” [4]. A criticism of this definition was its focus on the skill set of the individual without taking into account the integral role that characteristics of the health care system play in facilitating or impeding the individual’s successful navigation of health-related tasks [2, 4]. Subsequently, the WHO developed a toolkit in 2015 for HL strengthening in low- and middle income countries based on a conceptual framework that includes both the individual and health systems characteristics that influence the HL of an individual in a particular situation [8]. Tools to measure HL have been validated in several Asian languages [8–10] but limitations include weak theoretical grounding, culturally specific elements, and use of scales that require numeracy [11–13].

The Myanmar-Thailand border is home to migrant, ethnic minority and conflict-affected populations who are at risk for low HL and poor health outcomes [14] due to limited health care and educational access [15]. Prevalent low chronic disease knowledge among ethnic Karen farmers in this region [16–18] has been linked to adverse health outcomes [16]. In contrast, a study conducted in Shoklo Malaria Research Unit (SMRU) clinics providing antenatal care (ANC) in migrant and refugee communities in the same area did not show an association between delivery outcomes and literacy and demonstrated significant improvement in health outcomes over the study period despite persistently low levels of literacy [19].

In the absence of validated measures in Thai or Karen languages, these studies used locally tailored approaches to assess knowledge and literacy [19]. The health knowledge evaluation in the chronic disease studies correlates with oral HL [2], as the surveys orally assessed the respondents’ ability “to gain access to, understand and use information in ways which promote and maintain good health”. In the studies done by SMRU, literacy was tested by asking participants to read written health messages typical of those that they would encounter at local clinics, with results that approximate print HL [2]. Though neither tool is formally validated against a gold standard, as currently none exists, a strength of these studies is the local contextualization of the tools used, reflecting the health information challenges the participants are likely to face. This touches on the key domain of health system characteristics [8].

Recent research has assessed challenges to effective health messaging in migrant populations [20–22], and efficacy of visual health messaging to populations with low literacy [1, 23, 24], but most studies have been conducted in high-income countries. Posters as health promotion tools are appealing as they are a low-cost intervention that can potentially reach a wide audience [25], and are commonly used in health promotion programs in low-income countries. However, a 1989 study in India found that posters were often misinterpreted because of specific local understandings of the images, and piloting of posters in target communities was recommended [26].

Public health programming through visual health messaging has met with considerable obstacles along the Myanmar-Thailand border. In response to high rates of neural tube defects in populations seeking ANC at SMRU clinics, a campaign to promote preconception folate [27] was launched, including focus group discussions (FGD), workshops, and colorful multilingual posters and pamphlets. Evaluation 18 months later showed that preconception folic acid uptake in the community remained less than 2%. Themes derived from FGD included concern that low literacy diminished the impact of written educational materials [28].

**Fig 1:**
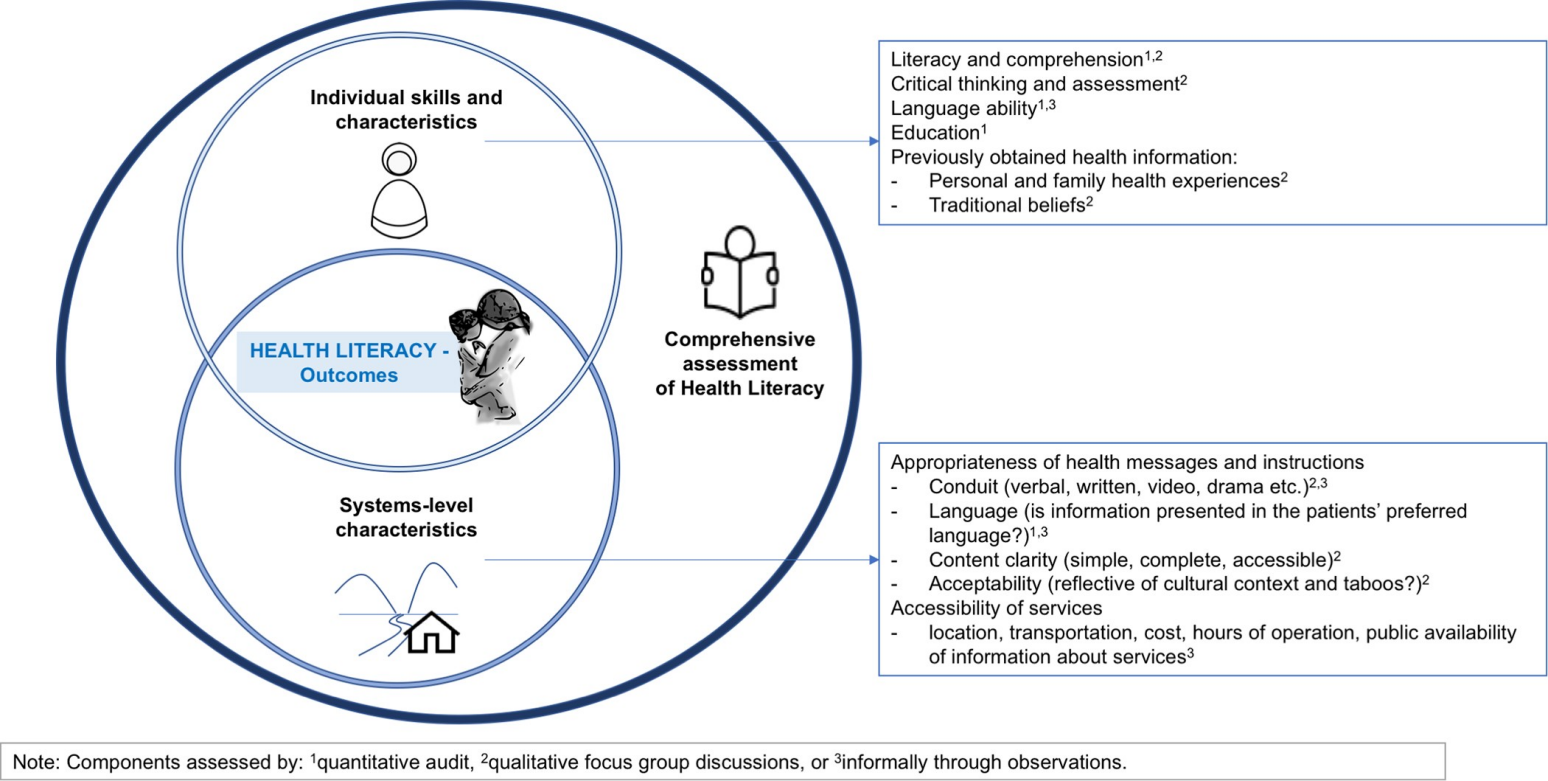
Conceptual framework for health literacy: The interaction of individual and systems domains.

Using the folic acid campaign as a case study, this study employs a mixed-methods design to paint a comprehensive picture of HL in this setting (Fig 1). We approach HL from (1) the individual skills domain by assessing degree of HL in the patient population and comparing simple demographic measures (reported literacy and grade attained in school) with clinically assessed HL in this population; and (2) from the health systems domain by using FGD to elicit the opinions of literate and illiterate ANC attenders about the clarity and appeal of public health posters used in the campaign. In our conceptual model, these two domains intersect to determine the HL of an individual and influence health outcomes. Findings from this study will inform future health messaging and advocacy along the border with implications for similar populations with low HL.

## Methods

### Setting

SMRU has offered free ANC services along the Myanmar-Thailand border since 1986. This survey was conducted in two clinics serving migrant workers with limited access to the national health services in Thailand and Myanmar.

Commonly spoken languages include S’gaw and Pwo Karen, and Burmese, with a few women speaking other ethnic languages or Thai. The most common written languages are S’gaw Karen and Burmese. Pwo is infrequently used as a written language and only 3.4% (39/1149) of literate women reported reading Pwo Karen in the previous survey [19].

Since 2010, data on self-reported literacy in any language is routinely gathered from women at their first ANC visit. Because formal education in these communities has been limited [15], all women receiving care at SMRU clinics are effectively treated as illiterate, using verbal explanations, demonstrations, and reliance on the teach-back method of instruction [29] in lieu of written instructions.

### Study design

This study employed mixed methods through a sequential explanatory design [30]. A cross-sectional clinical audit evaluated HL of women presenting to SMRU ANC clinics. This was followed by FGD to explore women’s understanding of health messages at the clinic, using posters promoting preconception folic acid to stimulate discussion [28].

#### Assessment of academic history and health literacy

The HL assessment was carried out between 24 March and 13 June 2017, resulting in data on 525 women. All women presenting on a given day were assessed for reading fluency and comprehension, and assessments were carried out at the two clinics over two months. Women’s pregnancy records were marked when they had completed testing to avoid duplication.

This clinical assessment of HL was preferred over translation of other common HL tools because of the extensive history with this method at SMRU [19, 31] and the poor relevance or acceptability of available HL tools in the target population (S1 Table).

Testing of reading ability was done one-on-one with women in the ANC waiting room by a researcher fluent in each of the languages. Women were asked to read aloud four written sentences that have been used in multiple surveys in this setting (1995–1997 [31], 2003 and 2008 [19]). The sentences are representative of common messages delivered by health workers at SMRU and other border health facilities and were available in Burmese, S’gaw Karen, English, and Thai (S1 Fig). Accuracy of the sentences was re-checked prior to start of the study and women chose their preferred language.

Comprehension was assessed by asking the patient to explain the sentence, mirroring the teach-back method routinely used in this setting. The original survey from the 1990’s included this comprehension component for one statement, whereas the later surveys focused on reading fluency alone.

At the end of the present assessment, women were given two scores, one for reading fluency and one for comprehension, each scored as full ability (2), partial ability; ((1) if some words could be read but not all, or if the participant could only partially explain the sentence); or inability (0). Women who did not attempt the test because of total illiteracy were given a score of 0. A composite score was calculated using the following formula:

compositescore=readingscore×comprehensionscore

This gave possible scores of 0, 1, 2, and 4, and this was dichotomized to low HL (0 or 1) or adequate HL (2 or 4). Multiplication (rather than addition) was chosen to calculate the composite score in order to reflect that a score of 0 for either component represented low HL.

Academic history was assessed by self-reported grade completed in school. The Myanmar school system has major exams at Grade 4, Grade 8, and Grade 10, and these milestones were used as *a priori* cut-offs for analysis.

#### Focus group discussions

FGD were conducted at SMRU ANC clinics with women who fit the inclusion criteria (healthy pregnant women presenting for routine ANC, fluent in Burmese or S’gaw Karen). Women who were acutely unwell or unable to participate in the language of the discussion were excluded.

Consenting participants were grouped based on shared characteristics: self-reported literacy, parity (nulliparous vs. parous) and preferred language (S’gaw Karen or Burmese) to create the optimal setting for free discussion and to allow for comparisons across groups. In the local culture, younger women without children (nulliparous) will normally defer to women with children (parous) in a group discussion and less educated women tend to defer to more educated women. Previous studies in this setting have found 4–8 participants per FGD ideal, with saturation of key themes reached with 7–10 FGD [32, 33].

Eight FGD of one hour each were carried out over three weeks in a private area. Each participant was only included in one discussion and a minimum of two research staff were present to conduct FGD. One facilitator, fluent in either Burmese or S’gaw Karen, led the FGD while a second facilitator took notes. FGD facilitators underwent study-specific training prior to facilitation including the purpose of the study, the meaning of the posters and FGD guide questions, as well as general information about active listening and facilitation. There were a total of four FGD facilitators involved in the study (PM, NP, KW, MP). All are fluent in S’gaw Karen and three are fluent in Burmese. Two (NP, PM) were experienced FGD facilitators from previous SMRU studies. KW is a trained counsellor, and PM is a professional medical translator. KW and PM received additional theoretical training and mentoring in FGD facilitation before taking a facilitation role.

Participants were shown three health posters relating to preconception folic acid, with varied written text (in Burmese, Karen, and Thai) and imagery (S2 Fig). Facilitators asked FGD participants their impression of the posters following a semi-structured guide developed by three of the authors (MEG, PM, AH) and reviewed by an additional author (NP) for local acceptability (S1 File). Questions focused on clarity, visual appeal, and perceived meaning of each poster, with clarifying prompts to identify sources of confusion or offense. After each FGD, researchers debriefed and discussed impressions of the discussion and identified potential themes and possible ways to improve the posters or the discussion flow. Discussions were audio-recorded and subsequently transcribed into English for analysis. Translation was done by facilitators and a professional service, and transcripts were cross-checked with notes taken during FGD.

### Analysis

#### Quantitative

Data were analyzed using Stata version 15 (StataCorp, CollegeStation, Texas). Continuous, normally distributed data were summarized as means with standard deviation or range; continuous, non-normally distributed data with median and interquartile range; and binary data with proportions or percentages and 95% confidence intervals (CI). Trends across ordered groups were analyzed using Cuzik test and Kruskal-Wallis test was used for comparing continuous variables across unordered groups. Sensitivity, specificity, negative predictive value (NPV), and positive predictive value (PPV) of potential predictive variables for the binary variable of HL were calculated. Receiver operating characteristic (ROC) curves were generated and areas under the curve (AUC) were compared.

#### Qualitative

Three researchers reviewed the transcripts and independently performed open coding using NVivo software. MEG coded all of the FGD transcripts, while PM and WJ coded selected FGDs. Researchers met to discuss codes and developed a unified codebook based on this discussion. MEG proceeded to re-code data according to the codebook and performed a preliminary thematic analysis. Themes were finalized after discussion of preliminary findings among MEG, WJ, AH, CA.

### Ethics statement

The clinical audit component of this study was undertaken to improve clinical care and did not require ethical approval. ANC attendance at SMRU clinics is voluntary and all women were informed that participation in the audit was voluntary. The qualitative component was reviewed and approved by the Research Ethics Committee of Chiang Mai University (FAM-2560-04815), the Oxford Tropical Research Ethics committee (OxTREC 524–17), and the Tak Community Advisory Board (TCAB 2017429/TCAB-04). Verbal consent was chosen to minimize stigma for illiterate participants, was documented by audio recording, and this was approved by the two ethics committees and highly recommended by the community advisory board.

## Results

### Health literacy

Baseline demographics of assessed women are summarized in Table 1. Overall, median gravidity was two and 38% of women were nulliparous. About half of the women were ethnically Karen, and almost all women reported Myanmar as their country of birth, while more than 40% were residing in Thailand at the time of the survey. Only 171/525 (32.6%) of women in this cohort completed 4^th^ grade or above, and 20/525 (3.8%) had completed high school (Grade 10).

**Table 1:** Demographics of all women included in the cross-sectional clinical audit of health literacy.

Characteristic		All women (n = 525)
**Age**^**1**^		26 ± 7 [14–46]
**Teenagers**^**2**^		20% (103)
**20–29 years**^**2**^		50% (261)
**≥30 years**^**2**^		31% (161)
**Nullipara**^**2**^		38% (200)
**Gravidity**^**3**^		2 [1–11]
**Parity**^**3**^		1 [0–9]
**Ethnicity**^**2**^	**Burman**	41% (213)
	**S’gaw Karen**	33% (172)
	**Pwo Karen**	19% (101)
	**Other**	7% (39)
**Smokers**^**2**^		8% (44)
**Country of residence (Myanmar)**^**2**^		58% (303)
**Country of birth (Myanmar)**^**2**^		98% (513)
**Grade completed in school (n = 525**	**0**	42% (221)
	1–3	25% (133)
	4	13% (66)
	5–7	11% (58)
	8	3% (15)
	>8	6% (32)

Data are presented as mean ± SD [range] ^1^

as percentage (number) ^2^

or as median [range] ^3^

Overall, 331/525 (63.1%) women reported that they could read, but only 194/525 (37.0%) had adequate HL when tested [Table 2]. Comprehension was generally lower than reading fluency, with 11 participants able to read entire sentences but completely unable to explain their meaning (S2 Table).

**Table 2:** Health literacy scores for women included in the cross-sectional clinical audit.

Characteristic	Group (n)	Proportion low literacy, % [95%CI]	P value*
**Spoken Language (n = 460)**	**Burmese (231)**	57 [50–63]	0.017
	**S’gaw Karen (155)**	66 [58–73]	
	**Pwo Karen (69)**	75 [63–84]	
	**Other (Mon, Thai, Hmong, Rakhine) (5)**	60 [17–92]	
**Reading language (n = 275)**	**Burmese (230)**	38 [32–44]	0.311
	**S’gaw Karen (40)**	43 [28–58]	
	**Thai (5)**	20 [2–75]	
**Grade attained in school (n = 525)**	**0–3 (354)**	85 [81–88]	<0.001
	**4–7 (124)**	22 [15–30]	
	**≥8 (47)**	6 [2–18]	

* p value for median composite score

HL was lower among Karen speakers than among Burmese speakers. There was no difference in HL when women were grouped by preferred reading language, but this data is limited to those who chose a preferred reading language, eliminating those with very low literacy. All Burmese speakers who attempted the test chose to read in their primary spoken language, Burmese. As testing was not offered in Pwo Karen, Mon, Rakhine or Hmong, all women in these groups were tested in a second language of their preference. Women who spoke S’gaw Karen were split between reading Burmese (36/76), S’gaw Karen (36/76) and Thai (4/76), and HL was similar between these groups (Table 2).

#### Predictors of low health literacy

Low HL was found in 145/331 (43.8%) of women who reported being able to read and 186/194 (95.9%) of women who reported being illiterate. Reported illiteracy had a high specificity and PPV as a predictor of low HL in this population (Table 3) but was weak in terms of sensitivity and NPV. Exploratory analysis of grade attained in school as an alternative screening question for low HL in this population found that, using cut-points between grade 2 and grade 4, grade attained in school yielded higher sensitivity and NPV than reported literacy, while maintaining specificity >70%. Area under the ROC curve was highest using a cut-point of 3^rd^ grade.

**Table 3:** Predictors of low or adequate health literacy.

Clinical Variable	Sensitivity % (95% CI)	Specificity % (95% CI)	PPV % (95% CI)	NPV % (95% CI)	ROC AUC
**Reported illiteracy**	56.19 (50.66–61.61)	95.88 (92.04–98.20)	95.88 (92.04–98.20)	56.19 (50.66–61.61)	0.76
**Less than 2**^**nd**^ **grade education**	71.60 (66.41–76.40)	95.36 (91.38–97.86)	96.34 (93.17–98.31)	66.31 (60.43–71.83)	0.83
**Less than 3**^**rd**^ **grade education**	83.99 (79.58–87	85.57 (79.82–90.19)	90.85 (87.05–93.83)	75.80 (69.57–81.32)	0.85
**Less than 4**^**th**^ **grade education**	90.94 (87.31–93.80)	72.68 (65.84–78.82)	85.03 (80.88–88.58)	82.46 (75.91–87.84)	0.82
**Less than 5**^**th**^ **grade education**	95.77 (93.01–97.67)	46.91 (39.72–54.19)	75.48 (71.07–79.52)	86.67 (78.64–92.51)	0.71

Note: ROC AUC for grade as a continuous variable = 0.91. Abbreviations: CI confidence interval, PPV positive predictive value, NPV negative predictive value, ROC receiver operating characteristic, AUC area under the curve

### Focus group discussions

A total of 8 FGD were conducted, with 4 to 8 women per group and a total of 42 women included. They were grouped according to parity, literacy and language as described in Table 4. Themes and codes from analysis are summarized in Table 5.

**Table 4:** Characteristics of women participating in focus group discussions.

FGD	Language	Literacy	Parity	# of participants
**A**	Karen	Literate	Nullipara	5
**B**	Burmese	Literate	Multipara	5
**C**	Burmese	Literate	Nullipara	6
**D**	Burmese	Illiterate	Multipara	8
**E**	Karen	Illiterate	Multipara	6
**F**	Karen	Illiterate	Nullipara	4
**G**	Karen	Literate	Multipara	4
**H**	Burmese	Illiterate	Nullipara	4
**Total**				42

**Table 5:** Women’s perceptions of public health posters–themes and codes from focus group discussions.

Themes	Codes
**Relationship between literacy and comprehension**	
Illiteracy	• frustration and disempowerment, curiosity • looking at pictures only (without reading text) commonly led to misinterpretations • the poster concept should be simple and positive (taking pills for healthy baby)
Literacy	• reading text is the key to understanding the poster • the poster concept should include both positive and negative messages (taking pills for healthy baby, if not the baby will be abnormal)
Symbols	• “X” indicates “not taken” or “dangerous, you should not take it”
**Perceptions related to pills**	• What is it? (eg. OCP) • Beneficial, preventing congenital abnormalities • Dangerous, causing congenital abnormalities • Prevention vs. cure
**Timing and consistency of folic acid supplementation**	• Should take the pill regularly • Should take pill when pregnant (eg. start at first ANC visit) • Should stop pill before pregnancy (eg. OCP) • Calendar, moon, menses can represent time to take pills but need explanation, many possible misinterpretations
**Local phenomenology of congenital abnormalities**	
Looking	• A fetus can develop the negative characteristics of someone/something that a pregnant woman looks at
Karma	• Congenital abnormalities due to deeds of the fetus or mother in past lives) • Karma can be influenced by folic acid supplementation
Dietary taboos & heat	• Hot water, other dietary taboo or OCP cause heat inside uterus

#### Relationship between literacy and comprehension

Comprehension as it related to literacy and health messages was spontaneously discussed by the focus group participants (both literate and illiterate) and probed by the moderators. All agreed that literacy was an important mediator of understanding health messages. For illiterate participants, there was a theme of frustration and disempowerment in some comments about their inability to understand the posters. However, some expressed curiosity.

“I can’t read and don’t understand anything and don’t know what to say.” [Karen Illiterate Nullipara]

“What type of medication is it? I want to know what the writing says. Is the medication for the mother, baby, or is it a birth control? I think the medication is for the baby. But I don’t know about the writing.” [Karen Illiterate Nullipara]

Multiparity appeared to help women compensate for low reading ability. Illiterate women with previous pregnancy experiences were better able to interpret the posters than illiterate nulliparas, suggesting that knowledge transfer did occur during the experience of prior pregnancy, ANC, and delivery (often through SMRU clinics).

Several literate patients stated that the text was central to their understanding of the posters, and that they couldn’t think of how to better convey the message to an illiterate audience. One participant suggested that only a spoken explanation would communicate effectively to illiterate women.

“I would not be able to understand the poster by just looking at the pictures. I got the message by reading the writing.” [Karen Literate Multipara]

“For those who can’t read, only the talking explanation would do.” [Burmese Literate Multipara]

Despite higher comprehension of the health messages in the literate groups, some literate women still expressed significant misunderstandings including: that the pills depicted are oral contraceptive pills (OCP), that the pills were the cause of the fetal abnormalities, that folic acid supplementation is only needed during pregnancy, and that the pills prevented sexually transmitted infections. Other misinterpretations of the posters came up only in the illiterate groups (e.g., that the intended message is about healthy diet and exercise in pregnancy). A minority of the illiterate women demonstrated relatively accurate interpretations of the posters without explanation.

Although literate participants came from the same communities as illiterate ones, they had some misconceptions about their illiterate neighbors. For example, one literate Karen participant suggested that using symbols such as check marks and X’s would clarify for illiterate individuals which women in the posters were and were not taking folic acid. Both Burmese literate groups supported this idea. However, in both Karen illiterate groups women expressed confusion about the meaning of these symbols and some Burmese illiterate women interpreted the symbols to indicate whether the pills that the women were taking were safe or dangerous in pregnancy.

The most popular poster overall was the simplest (S2B Fig), which only showed one narrative: a woman taking preconception folic acid and having a healthy baby. This poster was described as “clear” and was especially favored among the illiterate groups. In contrast, a theme that emerged in the literate groups was a preference for the more “complete” information in Posters 1 and 3 (S2A and S2C Fig), which included both pictures of healthy infants and those with neural tube defects.

“I think posters 1 and 3 are a bit messy. There is too much information on the poster. They don’t look as clear as poster 2.” [Karen Illiterate Nullipara]

“It’s always best to fit the two forms of information.” [Burmese Literate Multipara]

#### Perceptions related to pills

Women in all groups discussed extensively what the pills in the posters were, their indications, and their effects.

One of the key intended messages in the posters was that lack of folic acid supplementation could lead to congenital abnormalities—a message understood by most participants. In 33 mentions of “not taking” the pills, every instance linked lack of pill-taking with risk for congenital abnormalities or poor fetal health.

“If you don’t take pill, the baby will be abnormal.” [Burmese Illiterate Nullipara]

However, out of 66 mentions of taking pills, 15 statements linked pill-taking to an adverse outcome. This interpretation that the posters were warning against taking unsafe medicines in pregnancy came up in half of the groups, including both Burmese and Karen, primipara and multipara, and interestingly both literate and illiterate women.

The misunderstanding was tied to confusion about what medicine was being depicted in the images on the posters. In Poster A (S2A Fig), two identical images were used—one to show folic acid being taken leading to a healthy outcome and the other (with an “X” over it) to indicate that not taking folic acid could lead to an unhealthy outcome. Some participants stated that these two identical images were depicting two different kinds of pills responsible for healthy and unhealthy outcomes, respectively.

“The first picture of medicine in the middle is for something that causes the baby to become sore/hurt. The second picture of medicine is for something to cure the baby’s sore until it’s cured permanently.” [Burmese Illiterate Multipara]

The most common misperception (expressed in 6 of 8 groups) was that the pills were OCP, considered dangerous because of their “hot” properties. This confusion was caused by associations between taking OCP and sexual intercourse (depicted in Posters 2 and 3 (S2B and S2C Fig). One literate patient even seemed to identify the pill as OCP from the text.

“It’s like a kind of medicine that applies heat inside the womb… it can give trouble to the nerves because the medicine is designed to take ONLY before the pregnancy period as prevention—a contraceptive pill. It says, ‘The medication or contraceptive pill that prevents any harm from nerves-damages and physical disabilities and should be taken only before the pregnancy period.’ When you are pregnant, you cannot take it.” [Burmese Literate Multipara]

Prevention was explicitly mentioned in both literate Burmese groups and alluded to in most of the other groups.

“It says, ‘The medication that prevents any harm from nerve damage and physical disabilities.’” [Burmese Literate Multipara]

However, women in 3 of the 8 groups expressed that the pills would be able to cure fetal abnormalities that had already developed in utero.

“If you have an abnormal baby, this pill will make the baby healthier.” [Karen Literate Multipara]

Though subtle, this difference between prevention and cure implies different timelines for folic acid initiation.

#### Timing and consistency of folic acid supplementation

“Taking regularly” was a phrase used in 6 of 8 focus groups. In all cases adherence to regular medication regimen was viewed positively and poor adherence was viewed as a cause of poor maternal and fetal health.

“I think if you take the pill regularly, you will have a healthy baby. And if you don’t take it regularly, you will get an abnormal baby like the one in the poster.” [Karen Illiterate Nullipara]

However, the exact timing of “regular” folic acid administration (5 mg tablets dosed weekly starting three months prior to conception) was only described in two of the groups (both literate). Conveying this message of timing to an illiterate audience was a major challenge encountered throughout the discussion. Possible images were presented to the groups as options to clarify the timeline, including a calendar, a moon, and an image representing menstruation, and none seemed to reduce the misunderstandings.

There was a strong mental association for many women between taking folic acid and an established pregnancy. Because the FGD participants started taking supplements after their first antenatal visit, they assumed that a woman taking folic acid must be pregnant.

“We think the three women were pregnant because all the patients who come to the clinic are already pregnant and they’re taking the same yellow pills. Therefore, we believe that these women are pregnant while taking the pills…just like us.” [Karen Illiterate Multipara]

#### Local phenomenology of congenital abnormalities

The etiology of congenital abnormalities was not probed specifically, but FGD participants spontaneously offered traditional and medical explanations for neural tube defects. Medical causes described by participants included taking pills irregularly, not at all, or of the wrong type.

Traditional explanations were described in 5 of 8 groups, and differed by language group. Predominant traditional explanations for the development of congenital abnormalities were looking at a person or thing with undesirable characteristics, karma, and heat.

*Looking.* The concept that a baby might develop the negative characteristics of someone or something that a pregnant woman looks at was mentioned in both literate and illiterate, primipara and multipara groups. Though this belief was mentioned in one Burmese group, it was stated explicitly and repeatedly in three of four Karen groups. Participants stated that this would prevent some women from looking at the posters with depictions of abnormal infants. Illiterate women were more reluctant to look at the image than literate women.

“I didn’t look at the abnormal baby in drawing. I didn’t dare to look at it because of my belief…I worry that my baby is going to look like someone I look at. So I tried to avoid looking at the abnormal baby picture.” [Karen Literate Nullipara]

*Karma.* Karma was described in detail by some of the Burmese literate multiparous participants, and both the deeds of the fetus or the mother in past lives were described as causes of congenital abnormalities.

“If the mothers are kind-hearted and caring, their babies are healthy; and if not, then their babies will be born with physical disabilities, because bad people from their past lives will conceive into the wombs and be born as unhealthy children.” [Burmese Literate Multipara]

One woman explained her own difficult past deliveries in the context of karma, but expressed hope that the folic acid pill might counteract negative karma to achieve a healthy outcome.

“Some of the babies are born from their past deeds–the cycle of rebirth–and when this meets with the effect of the medicine, it becomes easy to deliver the baby. As for me, when I was carrying my eldest son, my placenta stuck and the same went for the middle son because he was a vacuum delivery. I think the folic acid could make it easier for us to deliver the baby.” [Burmese Literate Multipara]

*Heat.* Both literate and illiterate Burmese multipara groups described heat as a cause of congenital abnormalities. For the literate group, the cause of heat in the uterus was OCP ingestion during pregnancy, whereas the illiterate women referred to drinking hot water or eating the wrong foods. Beyond hot water, no specific taboo foods were described.

“I guess that because the mother drinks hot water, the baby becomes like this.” [Burmese Illiterate Multipara]

## Discussion

Multiple individual and systems-level factors converge to determine HL, which can have an important influence on health outcomes. However, existing tools to assess HL are ill-suited to make a comprehensive assessment of HL in this migrant population with very low educational levels. This mixed methods study sought to describe HL in this clinical setting, revealing significant local barriers to conveying health messages.

The challenges to identifying an appropriate tool to test HL in this population (S1 Table) highlight the key contribution of health service characteristics to HL [2, 5, 6]. As SMRU and other clinics on the Myanmar-Thailand border rely heavily on verbal explanations and require relatively low HL for patients to access and navigate care, tools assessing ability to access health information on the internet, or ability to understand medication package inserts or labels would overestimate the difficulties a patient might encounter accessing care in this setting. On the other hand, a woman who would be found to have adequate HL by the measure used in this study might functionally have low HL if transplanted in another context. As a HL score is rarely a metric that travels with a patient from one clinical setting to another (as a lab or imaging result might), the search for a “gold standard” test of HL that is valid across all settings is perhaps misguided and likely impossible to achieve. Instead, a community or clinical-setting based multidimensional assessment, as presented here, is more likely to lead to actions (at the health service, public health, and education levels) that can improve health outcomes. Several such actions were identified through this assessment.

High levels of illiteracy persist among pregnant women seeking ANC at the SMRU clinics, comparable to previous surveys [19, 31], and FGD responses corroborated these findings, revealing very low comprehension of the health message in the illiterate groups and significant misunderstandings even among women who reported being literate. The current clinical marker used to screen for low HL (self-report of reading ability) was found to be highly specific (95.9%) but have very low sensitivity (56.2%) for identifying low HL. As the implications of failing to identify a woman with low HL due to an insufficiently sensitive test are greater than the implications of misclassifying a woman as having low HL, a test with high sensitivity and NPV is preferable. The exploratory analysis done here suggested that using reported grade attained in school, with a cut-off of completed 3^rd^ or 4^th^ grade, could significantly improve sensitivity while maintaining specificity. This remains a straightforward question to use in the context of busy ANC clinics.

The previously described improvement of birth outcomes despite low literacy in this setting [19] may be the result of a model of care in which local community members are trained to deliver care [34] and are responsive to the low HL and specific language needs of their home communities. However, this study suggests a ceiling to the health improvement that can be accomplished through this staff-intensive, clinic-based care. For health outcomes that require action by asymptomatic individuals, for example diabetics [16, 18] or women preconception [28], community-based public health interventions are needed. Impacting community level attitudes and practices without the use of billboards, posters, and flyers requires extensive organization and sustained funding.

Both the quantitative and qualitative components of this study highlighted areas where the folic acid campaign failed to account for specific local conditions, leading to systems-level barriers in effectively communicating with the target population. These communication barriers functionally reduced the HL of individuals targeted by the campaign in ways that standard patient education practices used in SMRU clinics (i.e. verbal explanations, teach-back method) do not. Literate women in the FGD stated that the written words were essential for their understanding of the posters, and the majority of illiterate participants could not grasp the posters’ meaning. As less than 50% of the target population are literate, this was a major obstacle. Though printed materials for the campaigns featured images, as well as text, these images (and others tested in this study) proved to be insufficient to convey the intended message.

Unexpected misconceptions discovered in the FGD highlight the challenge of communicating clearly with images and the absolute need for piloting of posters and flyers, even when developed by local health workers. These misconceptions arose when content presented in the health systems domain (e.g., images of the abnormal baby, women taking pills) met unanticipated preconceived associations (e.g., folic acid supplements and established pregnancy) or traditional beliefs (e.g., the danger of looking) in the individual domain (Fig 1). Preconceived associations misled both literate and illiterate participants, making them less likely to grasp the key message that folate should be taken prior to attempts to conceive. Illiterate women in particular were more reluctant to look at the traditionally taboo images, further decreasing their access to the health message.

Most women were able to correctly answer questions about folate supplementation by the end of the FGD, which ended with 5–10 minutes of explanation about the intended message. Though time-consuming and costly, verbal explanation used in conjunction with images remains an effective method of communication across groups with mixed HL, and should not be abandoned for more expedient methods in this and similar patient populations. Studies of video messaging strategies in communities with low HL also show promising results [35, 36].

Beyond the health care and public health domains, these findings highlight broader social determinants that influence both individual and systems-level HL domains. Persistently low HL can be explained largely by very low levels of education: less than a third of the women surveyed had completed 4^th^ grade and less than 4% had completed high school. Educational options have been chronically limited for communities living in conflict-affected areas of Myanmar and for migrant children in Thailand. Although legal reform has improved access to the Thai educational system for migrant children and national funding for education in Myanmar has improved, specific funding for ethnic and migrant educational systems is currently unstable and sustained through a patchwork of independent donor and government funding. Some 200,000 migrant children living in Thailand are estimated to be out of school [37] and at risk for poor health outcomes.

Despite the mixed methods approach, which allowed triangulation on key points between methods and researchers, there were limitations to this study. In the audit, there were no specific measures in place to prevent contamination (with literate participants priming illiterate ones). As illiteracy is normalized and there was no incentive for women to perform “well” on the test, such behavior was thought to be unlikely and was not observed. In addition, it is possible that there were women who were literate in Pwo Karen and not in the other languages who would then have been misclassified as illiterate. This number is expected to be very low based on previous data and would not affect ability to navigate health services as Pwo is not used as a written language in border health facilities. Finally, the decision to use a locally tailored tool rather than an internationally validated tool for HL assessment represents both a strength and a weakness, as previously discussed.

The complexity of communicating effectively to populations with low HL is often under-recognized and more work needs to be done to identify effective strategies. This study was deeply embedded in the local context, but the overall conclusions described here echo findings from a similar study in India [26] and are likely to be applicable to other marginalized, rural populations.

## Conclusions

HL is a multidimensional concept and its measurement should assess both individual and health systems domains. Low HL remains common among ANC attendees at rural clinics on the Myanmar-Thailand border and represents a significant barrier to effective communication of public health messages. Misunderstanding of posters was common, highlighting the importance of piloting posters before widescale implementation and the significant challenges facing health workers seeking to broadly distribute health information in marginalized communities with low educational levels. Staffing for health projects in populations with low HL should reflect the time needed for adequate verbal counselling.

## Supporting information

S1 Table Summary of barriers to the use of common health literacy assessment tools. (DOCX)

S2 Table Self-reported literacy vs tested reading and comprehension. (DOCX)

S1 Fig Statements used to test reading and comprehension in the health literacy assessment. (TIFF)

S2 Fig Posters used to facilitate focus group discussions. (TIFF)

S1 File Semi-structured guide for focus group discussions. (DOCX)
