# Supplementary material for: “I can’t read and don’t understand”: Health literacy and health messaging about folic acid for neural tube defect prevention in a migrant population on the Myanmar-Thailand border
Source: PLoS One. 2019 Jun 13;14(6):e0218138. doi: 10.1371/journal.pone.0218138 (PMC6564004; doi:10.1371/journal.pone.0218138)
Supplement: S1 Table — (DOCX) [file pone.0218138.s001.docx]

| **S1 Table. Summary of barriers to the use of common health literacy assessment tools.** | | | | |
| --- | --- | --- | --- | --- |
| **Tool Name** | **Translation in SEA settings** | **Strengths for use in study setting** | **Barriers to use in study setting** | **Citation** |
| Health Literacy Questionnaire (HLQ) | Chinese, Indonesian and Vietnamese | Recommended in the region, validated in low resource settings | Relies on scales of difficulty which have been found to have very low acceptability in this clinical setting and require extensive and difficult explanation. | WHO 2015, Ing 2017 |
| Health Literacy Survey tool (HLS-EU-47) | Indonesia, Kazakhstan, Malaysia,  Myanmar, Taiwan, and Vietnam | Validated in Myanmar | Validated Myanmar population with dramatically higher education than the study population. Relies on scales of difficulty which have been found to have very low acceptability in this clinical setting and require extensive and difficult explanation. | Duong 2017, Oo 2017, Ing 2017 |
| The Rapid Estimate of Adult Literacy in Medicine (REALM) | None | Greater ease of administration | Consists of technical medical terms that do not exist in the local languages. Relies on the premise that correct pronunciation indicates comprehension. | Elder, 2012 |
| Test of Functional Health Literacy (TOFHLA) | None |  | Contain core elements that not used in SMRU clinics (medication package inserts, medication labels) and/or are USA specific (Medicaid eligibility). | Jordan, 2011 |
| Newest Vital Sign (NVS) | None | Greater ease of administration | Core element is a USA nutrition label.  Proprietary. | Jordan, 2011 |
