# Supplementary material for: “I can’t read and don’t understand”: Health literacy and health messaging about folic acid for neural tube defect prevention in a migrant population on the Myanmar-Thailand border
Source: PLoS One. 2019 Jun 13;14(6):e0218138. doi: 10.1371/journal.pone.0218138 (PMC6564004; doi:10.1371/journal.pone.0218138)
Supplement: S2 Table — (DOCX) [file pone.0218138.s002.docx]

| **S2 Table. Self-reported literacy vs tested reading and comprehension.** | | | | | | |
| --- | --- | --- | --- | --- | --- | --- |
|  | | | **Tested Ability, n (%)** | | | **Proportion Low Health Literacy, % [95% CI]** |
|  |  |  | **None [0]** | **Partial [1]** | **Full [2]** |  |
| **Self-reported literacy** | **Literate** | Reading | 58/331 (17.5%) | 86/331 (26.0%) | 187/331 (56.5%) | 44 [39-49] |
|  |  | Comprehension | 102/331 (30.8%) | 85/331 (25.7%) | 144/331 (43.5%) |  |
|  | **Illiterate** | Reading | 173/194 (89.2%) | 9/194 (4.6%) | 12/194 (6.2%) | 96 [92-98] |
|  |  | Comprehension | 179/194 (92.3%) | 8/194 (4.1%) | 7/194 (3.6%) |  |
