# Supplementary figures and images for: “I can’t read and don’t understand”: Health literacy and health messaging about folic acid for neural tube defect prevention in a migrant population on the Myanmar-Thailand border
Source: PLoS One. 2019 Jun 13;14(6):e0218138. doi: 10.1371/journal.pone.0218138 (PMC6564004; doi:10.1371/journal.pone.0218138)

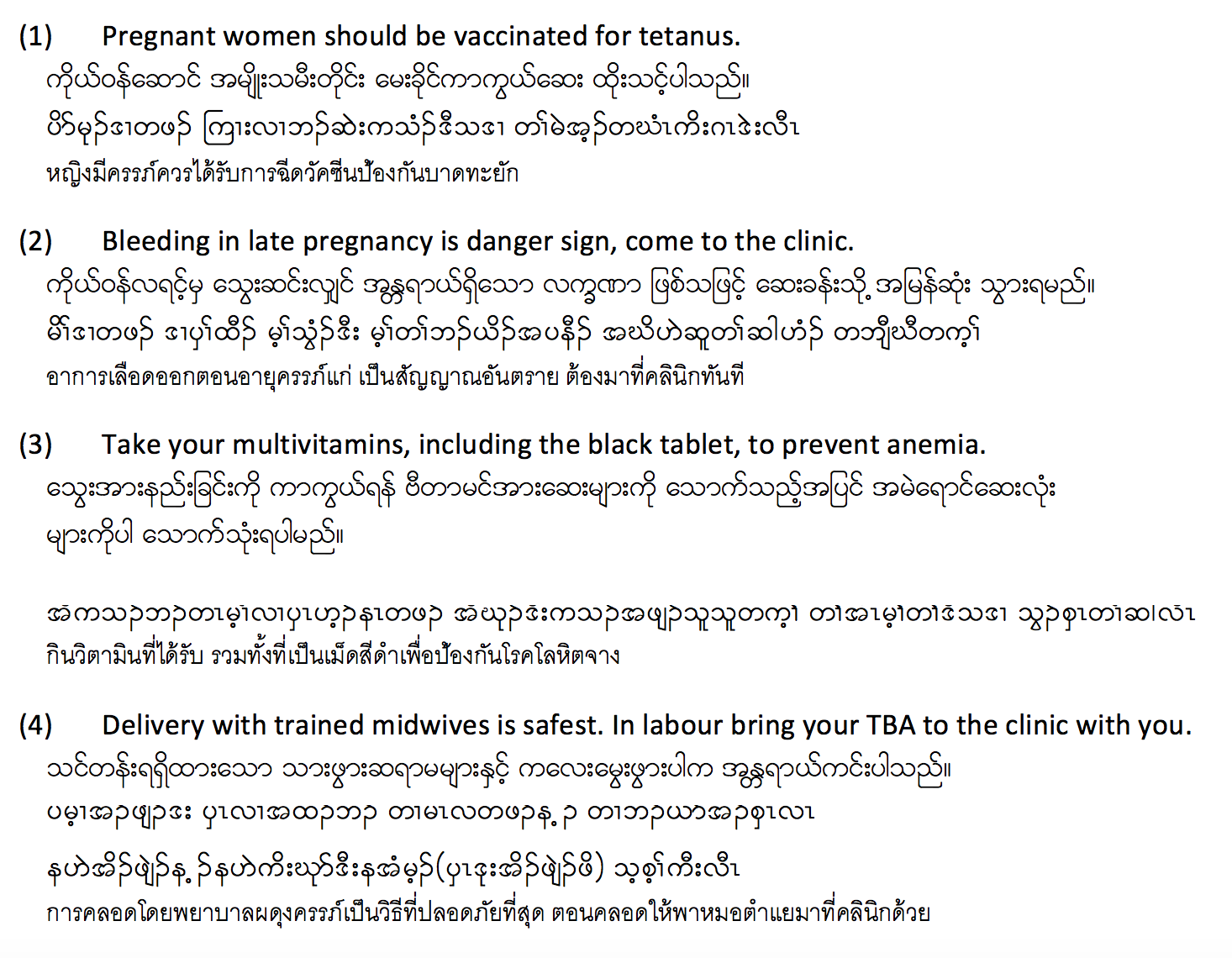

Supplement: S1 Fig — (TIFF) [file pone.0218138.s003.tiff]

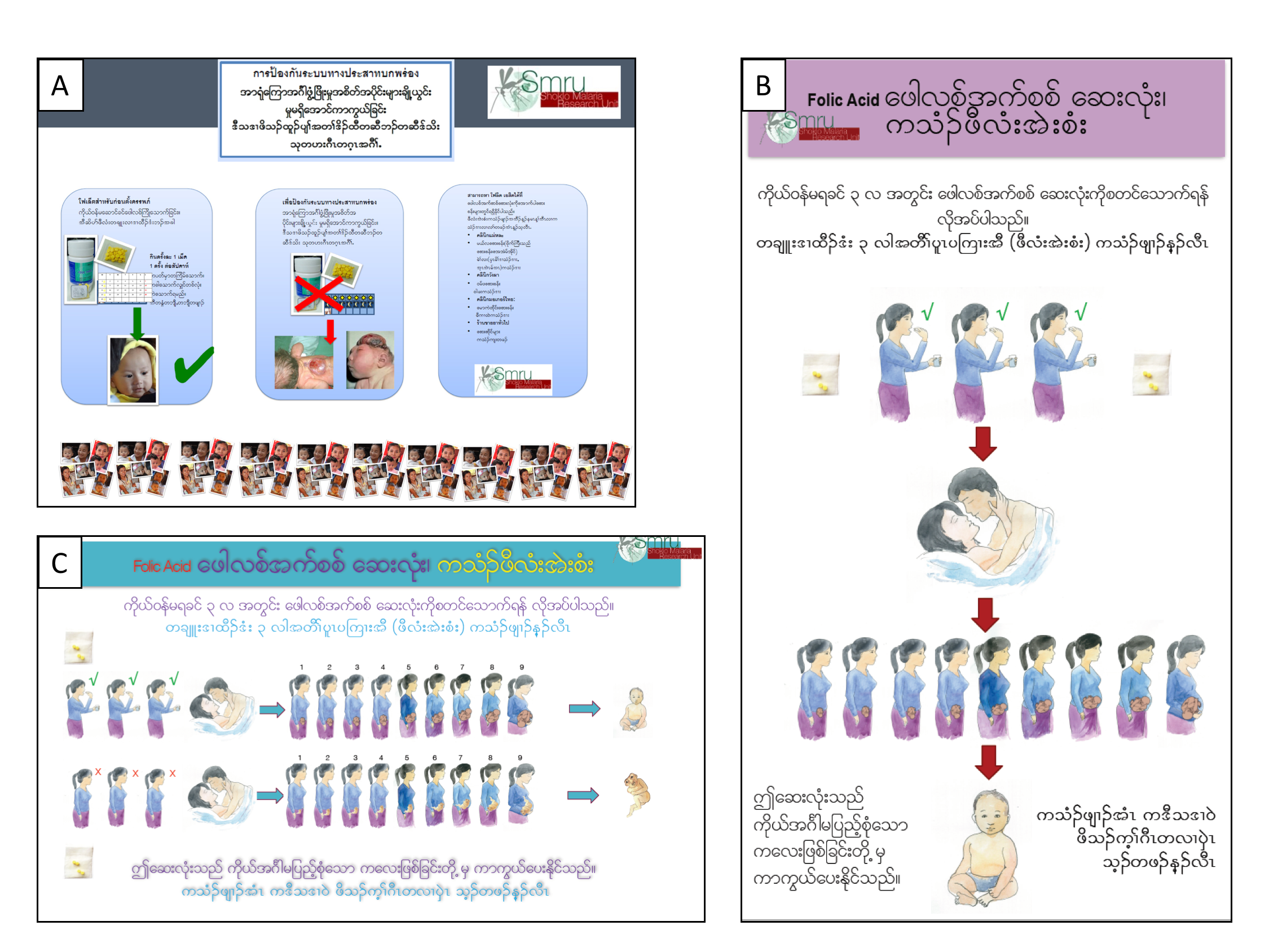

Supplement: S2 Fig — (TIFF) [file pone.0218138.s004.tiff]
