# Supplementary material for: “I can’t read and don’t understand”: Health literacy and health messaging about folic acid for neural tube defect prevention in a migrant population on the Myanmar-Thailand border
Source: PLoS One. 2019 Jun 13;14(6):e0218138. doi: 10.1371/journal.pone.0218138 (PMC6564004; doi:10.1371/journal.pone.0218138)
Supplement: S1 File — (DOCX) [file pone.0218138.s005.docx]

**Literacy and Health messaging**Shoklo Malaria Research Unit

**Focus Group Discussion Guide**

*Greeting: Posters will be on display in the room while taking informed consent, discussing the FGD procedure etc. Then, when ready to start FGD, facilitator hides the posters.*

*Ice breaker question:* Did you notice the posters we had on display? Can you describe anything from the posters?

Probe: Do you remember any words or images from the posters?

What were the posters about?

*The facilitator shows all 3 numbered posters and asks:*

1. Which poster catches your attention/eyes? Which is your favorite? Why? (takes a tally)

*Shows the favorite poster only*

1. What made you choose this poster?
2. What can you tell me about this poster? What does this poster mean to you? What message do you get by looking at this poster?
3. Is there anything you don’t like about this poster?

Probe: *Is there any reason you wouldn’t want to look at it?*

*Showing the other two posters one by one:*

1. What can you tell me about this poster? What does this poster mean to you? What message do you get by looking at this poster?
2. Is there anything you don’t like about this poster?

Probe: *Is there any reason you wouldn’t want to look at it?*

*Showing all 3 posters*

1. Do you have any additional suggestions of how to make these posters better?

Probe: *Which poster is more clear or easier to understand for you? Why?*
